# Itinerant and Correlated Nature of Altermagnetic MnTe Single Crystal Studied by Photoemission and Inverse-Photoemission Spectroscopies

**DOI:** 10.3390/ma18133103

**Published:** 2025-07-01

**Authors:** Kazi Golam Martuza, Yogendra Kumar, Hiroshi Yamaguchi, Shiv Kumar, Masashi Arita, Hitoshi Sato, Shin-ichiro Ideta, Kenya Shimada

**Affiliations:** 1 Graduate School of Advanced Science and Engineering, Hiroshima University, Higashi-Hiroshima 739-8526, Japan; m240915@hiroshima-u.ac.jp (H.Y.); jinjin@hiroshima-u.ac.jp (H.S.); idetas@hiroshima-u.ac.jp (S.-i.I.); 2 Department of Physics, Jahangirnagar University, Savar 1342, Dhaka, Bangladesh; 3 Research Institute for Synchrotron Radiation Science (HiSOR), Hiroshima University, Higashi-Hiroshima 739-0046, Japan; yogendra.phy@gmail.com (Y.K.); shivam.physics@gmail.com (S.K.); ariari@hiroshima-u.ac.jp (M.A.); 4 International Institute for Sustainability with Knotted Chiral Meta Matter (WPI-SKCM^2^), Hiroshima University, Higashi-Hiroshima 739-8526, Japan; 5 Research Institute for Semiconductor Engineering (RISE), Hiroshima University, Higashi-Hiroshima 739-8527, Japan

**Keywords:** MnTe, single crystal, altermagnetism, ARPES, IPES, density functional theory, electron correlation

## Abstract

Occupied and unoccupied electronic states of altermagnetic MnTe(0001) single crystals were studied by photoemission and inverse-photoemission spectroscopies after establishing a reproducible surface cleaning procedure involving repeated sputtering and annealing cycles. The angle-resolved photoemission spectroscopy (ARPES) exhibited a hole-like band dispersion centered at the $\bar{\Gamma }$ point, which was consistent with the reported ARPES results and our density functional theory (DFT) calculations with the on-site Coulomb interaction *U*. The observed Mn 3d↑-derived peak at −3.5 eV, however, significantly deviated from the DFT + *U* calculations. Meanwhile, the Mn 3d↓-derived peak at +3.0 eV observed by inverse-photoemission spectroscopy agreed well with the DFT + *U* results. Based on simulations of the spectral function employing an *w*-dependent model self-energy, we found significant relaxation effects in the electron-removal process, while such effects were negligible in the electron-addition process. Our study provides a comprehensive picture of electronic states, forming a solid foundation for understanding the magnetic and transport properties of MnTe.

## 1. Introduction

Altermagnetism is a newly identified magnetism with zero net magnetization in which magnetic sublattices are interrelated by crystal rotation symmetries, rather than by translation or inversion [1,2,3]. Altermagnetic materials have attracted much interest for their potential spintronic and thermoelectric applications [1,2,3]. Although hexagonal MnTe has recently been recognized as one of the most typical altermagnets, it has long been extensively studied for its unique transport properties closely coupled to magnetism. The crystal structure of MnTe is the NiAs-type structure (P6_3_/mmc), which is nonsymmorphic, with glide planes and screw axes [4,5,6]. The nonsymmorphic symmetry together with spin alignment is the ground of the altermagnetism of MnTe.

Neutron scattering measurements indicated that the magnetic moment of Mn is ferromagnetically aligned in the *c*-plane with a preferable direction of the *a*-axis, and the adjacent magnetic moments along the *c*-axis are aligned antiferromagnetically [7,8,9]. The Néel temperature is around 310 K, and the magnetization is 4.7 *μ*_B_, which is consistent with an effective magnetic moment of 5 *μ*_B_ for the Mn d^5^ electron configuration [7,8,9]. Density functional theory (DFT) calculations confirmed that the experimental magnetic structure was energetically the most stable in the ground state [10]. However, doping with 0.3% Li is sufficient to induce the *c*-axis component of the spin, and with 5% Li doping, it is completely along the *c*-axis [11].

MnTe is an insulator with an optical gap of 1.26–1.35 eV [12,13,14,15]. It is also a good thermoelectric material [16,17,18,19], and magnon drag plays an important role in the thermoelectric properties [20]. Magnon drag occurs when the magnons (spin waves), induced by a thermal gradient, transfers part of their momentum to the carriers (electrons or holes). The power factor of MnTe can be further increased by tuning the magnitude of the magnon–electron drag and the electrical conductivity by Cr doping [21]. The interplay between spin entropy, hopping transport, and magnon/paramagnon lifetimes can be utilized to develop high-performance spin-driven thermoelectric materials [21,22]. From a spintronic application point of view, the anisotropic magnetoresistance of MnTe should be noted [23,24], which is associated with the three-fold symmetry of the magnetic domains within the *c*-plane [25].

Since the recognition of altermagnetism as a new magnetic state, the occupied electronic structures such as electronic band dispersions have been extensively studied using angle-resolved photoemission spectroscopy (ARPES) on thin films grown by molecular beam epitaxy (MBE) [26,27,28] and cleaving bulk single crystals [29]. The exchange split band below the Néel temperature (*T*_N_) was observed [26], and three-dimensional band structures and constant-energy contours near the Fermi level have been confirmed to be consistent with the DFT calculations with on-site Coulomb interaction of *U* [27,29]. While the observed band structures from the MBE-grown thin films are considered to be consistent in the magnetic phase, *T*_N_ varies depending on the film thickness [26,28], likely due to substrate-induced strain. It is known that *T*_N_ increases with applied pressure [30,31].

To date, no attempts have been made to prepare well-defined clean surfaces of bulk MnTe single crystals using sputtering and annealing. Once it is established, one can examine bulk transport and magnetic properties as well as the electronic states on the same single crystalline samples by ARPES and angle-resolved inverse-photoemission spectroscopy (IPES). Furthermore, orientation-dependent surface-derived states could also be detected, as predicted by DFT [32].

While photoemission spectroscopy experiments on MnTe single crystals have been extensively conducted in recent years, investigations of the unoccupied states by IPES have thus far been limited to polycrystalline samples with clean surfaces prepared by in situ mechanical scraping [33,34,35,36]. It was reported that the exchange split Mn 3d↑ in the occupied state was located at −3.7 eV, and Mn 3d↓ in the unoccupied state was located at +2.9 eV, indicating an effective *U* value of *U*_eff_ = 6.6 eV [33,34,35]. The *U*_eff_ value was much larger than the exchange splitting of 4 eV in the standard LDA calculation [37,38]. Furthermore, a broad satellite structure was found at −8 eV, which is a direct evidence of strong electron correlation in Mn 3d states [33,34,35,36].

To take into account the electron correlation effect, configuration interaction (CI) cluster model calculations were performed, and an average of the Mn 3d–3d Coulomb interaction *U* = 4–5 eV and the ligand (Te)-to-the-Mn 3d charge transfer energy *Δ* = 0–2 eV were obtained [33,34,35,39]. Since *Δ* < *U*, MnTe is classified as a charge-transfer type insulator in the Zaanen-Sawatzky-Allen diagram [40]. The satellite structure was also found in the Mn 2p core-level spectra of MnTe [41]. While the CI cluster model calculations captured satellite structure in the single-particle excitation, it is not compatible with the itinerant nature of quasiparticles. To accurately describe the photoemission and inverse-photoemission spectra as single-particle spectral functions, many-body interactions must be incorporated in a manner consistent with the band theory.

In this study, by establishing a reproducible cleaning process of MnTe(0001) single crystal surface, we have investigated its occupied and unoccupied electronic states using photoemission and inverse-photoemission spectroscopies. The experimental results were compared with our DFT + *U* calculations, and we simulated single particle excitation spectra using a model self-energy function. We discuss the difference in the relaxation process via the p−d hybridization in the occupied and unoccupied states separated by the band gap.

## 2. Materials and Methods

### 2.1. Crystal Growth

A two-step melting procedure was used to grow high-quality MnTe single crystals. A quartz ampoule was filled with a stoichiometric amount of high-purity Mn (99.9999%, The Nilaco Corporation, Tokyo, Japan) and Te shots (99.9999%, The Nilaco Corporation, Tokyo, Japan), then the ampoule was sealed at a pressure of 4 × 10^−3^ Pa. The sealed quartz ampoule was heated to 1273 K gradually (50 K/h), maintained there for 72 h, and then cooled at 25 K/h to room temperature in an electronic furnace FUM312PC (Advantec Toyo Seisakusho Kaisha, Ltd., Tokyo, Japan). After this heat treatment, the sample was ground to ensure homogeneous mixing. The sample was finally put in a conical alumina crucible and sealed in a quartz ampule at a pressure of 2.8 × 10^−3^ Pa. The ampule was gradually heated to 1273 K and held there for 30 h. It was then heated to 1473 K and held for 24 h. Finally, the sample was gradually cooled to 973 K at a rate of 5 K per hour, then annealed for 12 h at 973 K before cooling to room temperature at a rate of 8 K/h.

### 2.2. XRD and EPMA

Powder X-ray diffraction (XRD) measurements with Cu K*α* radiation (*λ* = 1.54 Å) were performed on the as-grown MnTe sample with a SmartLab SE (Rigaku corporation, Tokyo, Japan). To analyze the XRD data, we referred to the crystallographic database for hexagonal MnTe (space group P6_3_/mmc, No. 194; JCPDS card # 18-0814) and cubic (pyrite-type) MnTe_2_ (space group $Pa\bar{3}$ , No. 205; JCPDS card # 18-0813). The lattice parameters were determined by the Rietveld refinement of the XRD patterns using the Fullprof software (version: December 2023) package. Pseudo-Voigt functions were used to fit the data based on *χ*^2^ minimization. To improve the numerical stability and convergence, the contribution from the Cu K*α*_2_ satellite line was subtracted from the XRD data prior to the refinement calculations. The elemental composition of the samples was examined using an electron probe micro-analyzer (EPMA) JXA-iSP100 (JEOL Ltd., Tokyo, Japan).

### 2.3. Clean Surface Preparation and Characterizations

**Figure 1:**
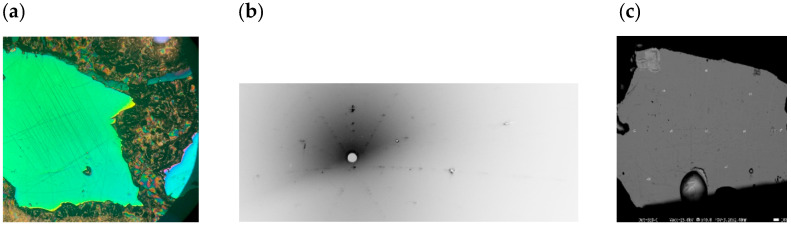
(**a**) Mechanically polished the single crystals of the MnTe(0001) plane. (**b**) Laue diffraction patterns from a (0001) *c*-plane displaying hexagonal symmetry. (**c**) Scanning electron microscope (SEM) image with points in white selected for EPMA analysis.

We cut and mechanically polished the single crystals to reveal the (0001) plane shown in Figure 1a. Figure 1b shows the Laue diffraction patterns from a (0001) *c*-plane displaying hexagonal symmetry.

The cleaning procedure of the MnTe(0001) surface was performed in three stages. To remove the surface contamination from the sample, argon ion sputtering was performed at beam energies of 1, 1.5, and 2 kV beam energy, followed by subsequent annealing at 523, 603, 633, and 673 K. To achieve an ordered and smooth surface, subsequent annealing is required. After every cycle of sputtering and annealing, we performed Auger electron spectroscopy (AES) measurements to check the surface cleanness and composition. The atomic concentration of an element (a) on a sample with n elements can be determined using the following equation, $X_{M}=\frac{N_{M}}{\sum_{i=1}^{n}N_{i}}=\frac{I_{M}/S_{M}}{\sum_{i=1}^{n}I_{i}/S_{i}}$ , where $I_{M}$ and $S_{M}$ are the Auger spectral intensity and relative sensitivity factors for element M, respectively. For percentage atomic concentration, $X_{M}$ × 100% was used in our calculations.

We optimized the cleaning conditions and finally obtained a clean surface by sputtering the sample at 2 kV beam energy for 5 min, followed by annealing at 673 K for 60 min. After each sputtering–annealing cycle, we ensured that the surface was clean and ordered.

### 2.4. Photoemission Spectroscopy and Inverse-Photoemission Spectroscopy

Angle-resolved/angle-integrated photoemission spectroscopy measurements have been performed on the linear undulator beamline (BL–1) of HiSOR, Hiroshima University [42,43] using a hemispherical electron-energy analyzer A-1 (MB Scientific AB, Uppsala, Sweden). The energy resolution was set at 20–50 meV. The ARPES data were taken at *hν* = 117 eV with the *p* polarization geometry. We set sample temperature at 20 K, and the base pressure of the main chamber was 3.6 × 10^−9^ Pa.

The resonant inverse-photoemission (RIPES) spectra of MnTe were taken with tunable photon energy mode. The light emitted from the sample was monochromatized by nonperiodic spherical gratings with the nominal line densities of 1200 lines/mm and magnified by the triple multichannel plates in front of a one-dimensional position-sensitive detector [36]. The sample was cooled using He closed cycles cryostat down to 20 K. The energy resolution was about 0.8 eV. The base pressure of the main chamber was 2.2 × 10^−9^ Pa. For RIPES measurements, we obtained a clean surface by Ar sputtering only because there is no heating stage available in the IPES system. However, we confirmed by AES measurement that Ar sputtering is effective to remove surface contamination, and it does not change the chemical composition of the surface.

### 2.5. Density Functional Theory Calculations

The electronic structure calculations for MnTe were performed using the full-potential linearized augmented plane-wave (FLAPW) method, as implemented in the FLEUR code (MaX Release 6.0) [44]. The basis set consisted of augmented plane waves in the interstitial region and atomic-like radial functions, along with their energy derivatives, inside the muffin-tin spheres. A maximum angular momentum cutoff of lmax = 12 was used, and local orbitals were included for Mn (3s, 3p) and Te (4d) states. Brillouin zone sampling for self-consistent calculations used a 14 × 14 × 14 *k*-point grid, and a separate path with 960 *k*-points was defined for band structure calculations.

To describe the exchange-correlation potential, both the generalized gradient approximation (GGA) formulated by Perdew, Burke, and Ernzerhof (PBE) [45] and the local density approximation (LDA) [46] were employed. To better account for the ground state properties such as magnetic moment and insulating ground state, we have employed the DFT + *U* formalism. Without the *U* value, one cannot explain band gap [37,38] and other ground state properties [14]. It was previously clarified that the lattice constants were reproduced for *U* = 2–3 eV and the Néel temperature for *U* = 4.5 eV [14]. We performed DFT + *U* calculations with varying *U* values, and confirmed that the band gap disappears at *U* = 0 eV and the magnitude of exchange splitting in the Mn 3d state increases with increasing *U*. The on-site Coulomb interaction was treated using the rotationally invariant approach with effective Hubbard parameters of *U* = 6.2 eV alongside a Hund’s exchange parameter of *J* = 0.86 eV referring to the previous calculation, as these parameters could reproduce experimental magnetic moment [47]. 

Spin–orbit coupling (SOC) effects were included self-consistently to capture relativistic interactions. We observed that the inclusion of SOC lifts the degeneracy of the energy bands at the A point and reduces the band gap by about 0.3 eV. In addition to band structure calculations, the total and orbital-resolved densities of states (DOS) were evaluated to provide a detailed understanding of the orbital contributions, particularly from Mn 3d and Te 5p states, near the Fermi level.

The structural parameters, including the lattice constants, were taken from Rietveld refinement of powder X-ray diffraction (XRD) data using the FullProf suite [48], ensuring that the theoretical model accurately reflects the experimental crystallography. We have also assumed an antiferromagnetic (AFM) configuration of MnTe obtained from experiments.

### 2.6. Spectral Function and Model Self-Energy

The single-particle excitation spectrum $A_{\sigma }\left(k,\omega \right)$ is given by the imaginary part of the single-particle Green’s function $G_{\sigma }\left(k,\omega \right)=1/\left[\omega -\omega_{k,\sigma }^{0}-\Sigma_{\sigma }(k,\omega )\right]$ :

$$A_{\sigma }\left(k,\omega \right)=-\frac{1}{\pi }\frac{Im\Sigma_{\sigma }(k,\omega )}{{\left[\omega -\omega_{k,\sigma }^{0}-Re\Sigma_{\sigma }(k,\omega )\right]}^{2}+{\left[Im\Sigma_{\sigma }(k,\omega )\right]}^{2}}$$

where $\omega_{k,\sigma }^{0}$ is the one-electron energy for wave vector *k* and spin *σ*. The real part of the pole of the spectral function, given by $\omega -\omega_{k,\sigma }^{0}-Re\Sigma_{\sigma }\left(k,\omega \right)=0$ , corresponds to the quasiparticle energy, while its imaginary part $Im\Sigma_{\sigma }(k,\omega )$ determines the quasiparticle lifetime (natural linewidth). Consider adding a hole to an initial *N*-particle state through photoexcitation, resulting in an (*N*−1)-particle state. Let $E_{N}^{0}$ be the energy of the initial state and $E_{N-1}^{0}$ the energy of the (*N*−1)-particle state without many-body relaxation. According to the Koopmans’ theorem, the one-electron energy is ${\omega_{k,\sigma }^{0}=E_{N}^{0}-E}_{N-1}^{0}$ . However, the actual (*N*−1)-particle final state undergoes energy shift (relaxation) due to many-body interactions, and the added hole can be regarded as a quasiparticle with finite lifetime. If the observed quasiparticle energy is denoted $\omega_{k,\sigma }$ , we have:

$$\omega_{k,\sigma }=E_{N}^{0}-E_{N-1}^{*}(\omega_{k,\sigma })=E_{N}^{0}-E_{N-1}^{0}+\left[E_{N-1}^{0}-E_{N-1}^{*}(\omega_{k,\sigma })\right]=\omega_{k,\sigma }^{0}+Re\Sigma (\omega_{k,\sigma }),$$

where the real part of the self-energy $Re\Sigma (\omega_{k,\sigma })=E_{N-1}^{0}-E_{N-1}^{*}(\omega_{k,\sigma })$ represents the energy shift due to many-body interactions.

In this study, we simulate the *k*-integrated spectral function by employing a *k*-independent model self-energy *Σ*(*ω*) for angle-integrated photoemission and inverse-photoemission spectra:

$$A_{\sigma }\left(\omega \right)=∭{A_{\sigma }\left(k,\omega \right)\;d}^{3}k=-\frac{1}{\pi }\int \frac{\rho_{\sigma }\left(\omega^{\prime }\right)Im\Sigma \left(\omega \right)}{{\left[\omega -\omega^{\prime }-Re\Sigma (\omega )\right]}^{2}+{\left[Im\Sigma (\omega )\right]}^{2}}d\omega^{\prime },$$

where $\rho_{\sigma }\left(\omega^{\prime }\right)$ is the density of states for energy $\omega^{\prime }$ and spin σ without relaxation effects. Here, Σ(ω) represents the dynamical relaxation induced by the addition of a photohole, defining quasiparticle energy and lifetime. By neglecting the *k* dependence, we implicitly assume that the excitation is a local one and does not alter global charge distributions that are closely linked to the underlying crystal symmetry described by the space group. 

Here, we briefly descript the derivation of a model self-energy following our previous paper [49]. We consider the transition from the unperturbed state $\left.\left|\psi_{i}\right.\right\rangle$ to perturbed final state $\left.\left|\psi_{r}\right.\right\rangle$ of the (*N*−1)-particle system using the first-order perturbation theory with a time-dependent many-body interaction $H^{\prime }e^{-i\xi t}$ , where *ξ* is a characteristic relaxation energy and $H^{\prime }$ is the perturbation operator. The transition probability is given by: $p_{ri}={\frac{\tau^{2}}{\hbar^{2}}\left|\left\langle \psi_{r}|H^{\prime }|\psi_{i}\right\rangle \right|}^{2}\sin^{2}\left(\frac{\omega_{ri}-\xi }{2\hbar }\tau \right)/{\left(\frac{\omega_{ri}-\xi }{2\hbar }\tau \right)}^{2},$ with $\omega_{ri}=E_{r}-E_{i}$ . In the limit of τ/2ℏ→+∞ , $p_{ri}$ reduces to the Fermi's golden rule: $p_{ri}/\tau ={\frac{2\pi }{\hbar }\left|\left\langle \psi_{r}|H^{\prime }|\psi_{i}\right\rangle \right|}^{2}\delta (\omega_{ri}-\xi )$ . In the case of finite quasiparticle lifetime $\Delta T=\tau /p_{ri}$ , we approximate the $\sin^{2}\left(\frac{\omega_{ri}-\xi }{2\hbar }\tau \right)/{\left(\frac{\omega_{ri}-\xi }{2\hbar }\tau \right)}^{2}$ function by a Lorentzian function $\pi /\left[{\left(\frac{\omega_{ri}-\xi }{2\hbar }\tau \right)}^{2}+\pi^{2}\right]$ . This approximation is justified because both give $\left(2\hbar /\tau \right)\cdot \pi \delta (\omega_{ri}-\xi )$ in the limit of τ/2ℏ→+∞ . Taking the uncertainty principle ΔE·ΔT=ℏ into account, the natural linewidth ΔE is given by: $\Delta E=\frac{\hbar }{\Delta T}=-2Im\Sigma \left(\omega_{ri}\right)$ . By defining $V={\left|\left\langle \psi_{r}|H^{\prime }|\psi_{i}\right\rangle \right|}^{2}$ and η=2ℏπ/τ , the self-energy takes the following form: $\Sigma \left(\omega \right)=\frac{V}{\omega -\xi +i\eta }$ . If there are several excitations, we add them as $\Sigma \left(\omega \right)=\sum_{n}\frac{V_{n}}{\omega -\xi_{n}+i\eta_{n}}$ .

Then, we impose conditions $\left|\;Im\;\Sigma \left(\omega \right)\right|\to \;0\;\left(\omega \;\to \;0\right)$ and $\frac{d}{d\omega }{\left|Im\;\Sigma \left(\omega \right)\right|}_{\omega =0}=0$ near the Fermi level. These conditions ensure infinite hole lifetime at the valence-band top and positive spectral function near the Fermi level, respectively. The simplest model fulfilling these conditions is: $\Sigma \left(\omega \right)=\frac{A+Bi}{\omega -\xi +i\eta }\;+\frac{\;C+Di\;}{\;\omega +\xi +i\eta }\;\left(\xi >0,\;\eta >0\right),$ with real parameters *A*, *B*, *C*, *D*, *ξ*, and *η*. Ensuring the above conditions, we derive relations A=ξB/η=C=−ξD/η . Setting g=2ξB/η , we finally obtain: $\Sigma \left(\omega \right)=\frac{g\;\omega }{\left(\omega -\xi +i\eta \right)\left(\omega +\xi +i\eta \right)}$ [49]. Note that the self-energy expansion near the Fermi level ( ω~0 ) is consistent with the Fermi-liquid theory: $\Sigma \left(\omega \right)\approx -\lambda \omega -i\;\frac{2g\eta }{{\left(\xi^{2}+\eta^{2}\right)}^{2}}\omega^{2}$ , with a coupling parameter $\lambda =-\frac{\partial Re\Sigma \left(\omega \right)}{\partial \omega }\;{|}_{\omega =0}=\frac{g}{\xi^{2}+\eta^{2}}$ .

The introduced model self-energy assumes the electron–hole symmetry. A more general asymmetric functional form could also be considered. However, this significantly complicates the equation and has little impact on the spectral behavior near ω=−ξ , though it may affect the spectral details near the Fermi level. For insulators like MnTe, the self-energy within the band gap should be zero. We applied the model self-energy function to simulate the occupied and unoccupied spectral function, using parameter sets that were not necessarily identical. In this study, we found that the self-energy effect on the inverse-photoemission spectra (IPES) of MnTe is almost negligible, suggesting that g is sufficiently suppressed in the unoccupied region and choice of the *ξ* and *η* does not affect significantly the shape of the IPES spectral function.

## 3. Results

**Figure 2:**
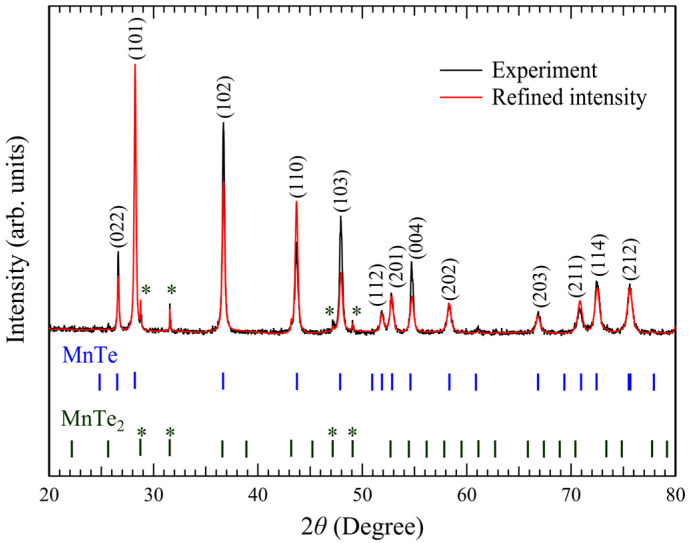
Rietveld refinement result of powder XRD pattern for the MnTe sample. Experimental data (black line) and Rietveld-refined fit (red line) assuming MnTe and MnTe_2_ phases. Vertical bars indicate the calculated diffraction peak positions for each phase. Asterisks (*) indicate distinct peaks attributed to the MnTe_2_ phase. MnTe_2_ phase fraction was estimated to be at most 4.8%.

Figure 2 presents the powder X-ray diffraction spectrum of MnTe single crystals. The Rietveld fitting of the dominant peaks is consistent with diffraction from the (022), (101), (102), (110), (103), and (202) planes of the hexagonal MnTe phase. After Rietveld refinement using FullProf software, the lattice parameters were *a* = *b* = 4.1485 Å, *c* = 6.7125 Å, *γ* = 120°, and *α* = *β* = 90°, which are consistent with the reference values [5,6,11,12]. The unit cell volume is evaluated to be 100.0440 Å^3^. The *c*/*a* value at the room temperature is 1.62, which is close to the ideal O_h_ coordination *c*/*a* = $\sqrt{8/3}$ = 1.63.

We have noticed a contribution from MnTe_2_, which is also found in previous publications [4,6,16,27]. It has been reported that in the oxidizing atmosphere, part of Mn is oxidated, and MnTe_2_ is created above 400 K [4,6]. However, all of the XRD peaks can be assigned to the MnTe and MnTe_2_, excluding other impurities such as MnO. We have estimated that the volume fraction of the MnTe_2_ impurity phase was at most 4.8% based on the two-phase Rietveld refinements, as shown in Figure 2. We assume that the MnTe_2_ detected in the XRD originates from grain boundaries. The electronic structure obtained from a single domain of MnTe(0001) phase, however, should not be affected by this minor phase. As described below, we confirmed that the ratio of Mn and Te atoms is 1:1 by various methods. Furthermore, the observed band structures are fully consistent with those of NiAs-type MnTe, which is completely different from MnTe_2_ with the pyrite-type structure ( $Pa\bar{3}$ ) [50,51].

Figure 1c shows the scanning electron microscope image of one of the three single-crystal samples that were cut from various ingot positions and used for the EPMA analyses. For each sample, we acquired compositions from twenty-four distinct locations. We obtained Mn ≈ 50.10% and Te ≈ 49.89% on average, indicating that the sample composition is almost 1:1.

**Figure 3:**
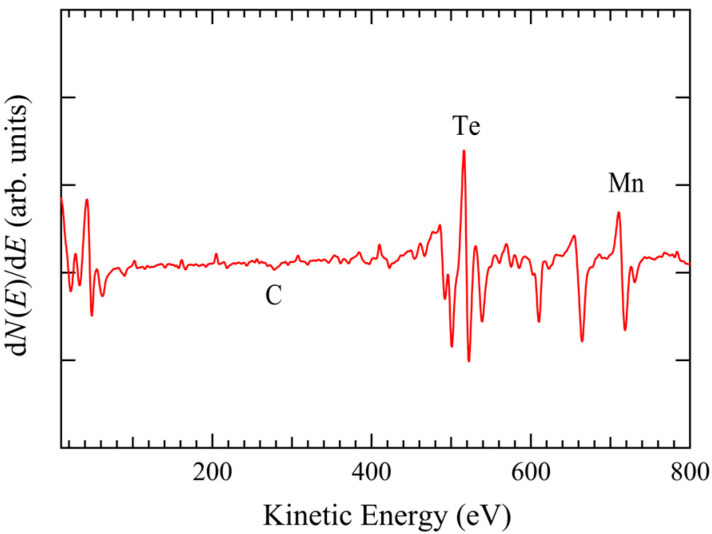
Final AES spectrum of the clean MnTe(0001) surface obtained after repeated cycles of sputtering and annealing.

Figure 3 shows the final AES spectrum of MnTe(0001) after repeated cleaning processes. The estimated Mn and Te atomic ratio, [Te]/[Mn], is 1.02, which is nearly 1:1. Here we should mention possible carbon (C) contamination of up to 3.8%, which was estimated from a shallow dip structure at the kinetic energy of ~290 eV, corresponding to the C KLL Auger line.

**Figure 4:**
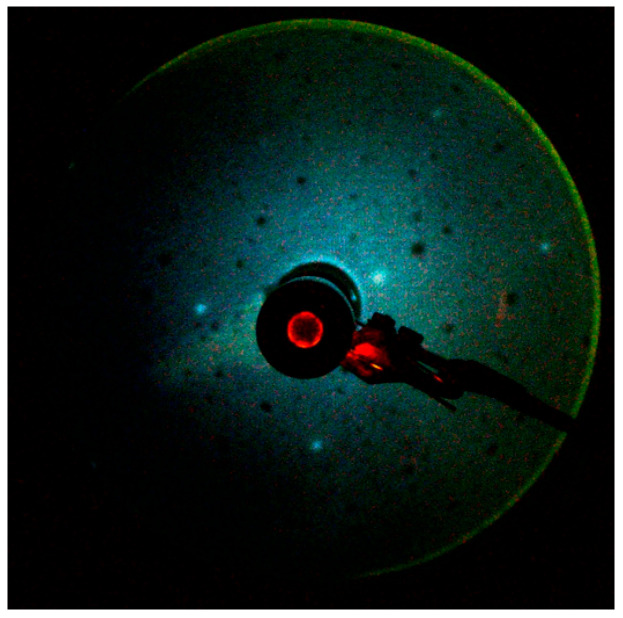
LEED image of the clean MnTe(0001) surface obtained after repeated cycles of sputtering and annealing. The incident electron kinetic energy was 42.2 eV.

Meanwhile, the low-energy electron diffraction (LEED) image taken at an incident electron kinetic energy of 42.2 eV (Figure 4) shows sharp six-fold symmetric spots. We do not see any superstructure due to adsorbed or segregated impurity atoms like C on the surface. The LEED image indicates a well-defined long range atomic order on the MnTe(0001) surface after cleaning process. We assume that a small amount of impurities on the surface, such as C, may contribute to additional ARPES linewidth broadening. However, the electron–impurity or electron–defect scattering is elastic and, therefore, does not alter the band structure [52].

**Figure 5:**
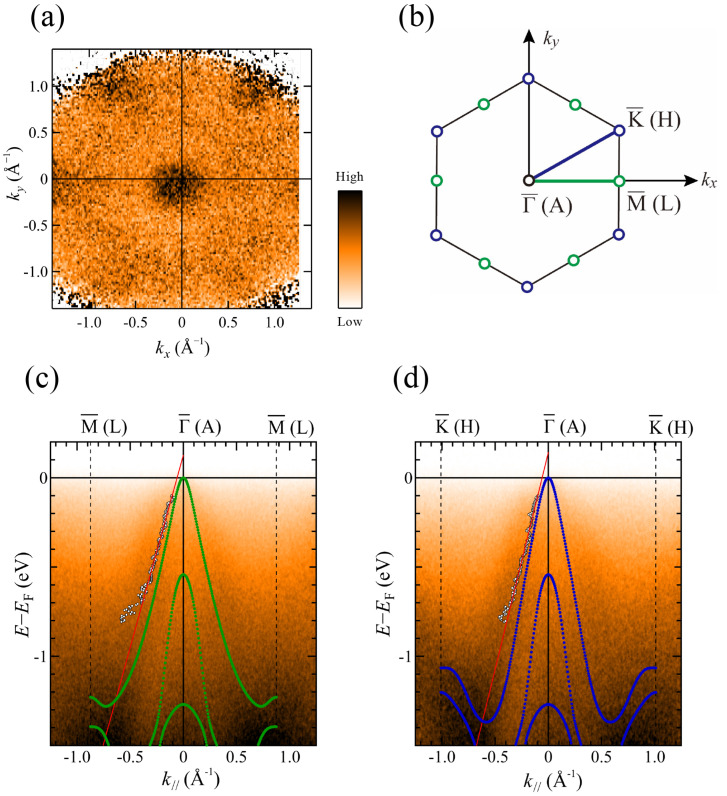
ARPES results for a MnTe(0001) single crystal measured at 20 K using photon energy *hν* = 117 eV in the *p*-polarization geometry. (**a**) *k_x_*–*k_y_* map near the Fermi level, obtained by integrating spectral intensity over the energy range from −0.1 eV to 0 eV. The *k_x_* direction corresponds to the $\bar{\Gamma M}$ direction. (**b**) Surface (bulk) Brillouin zone. ARPES intensity plots along (**c**) $\bar{\Gamma M}$ and (**d**) $\bar{\Gamma K}$ directions. Open circles indicate peak positions obtained by MDC fitting. Red lines represent fits to obtained data points from −0.6 to −0.1 eV. DFT + *U* calculations along the AL (green circles in (**c**)) and AH (blue circles in (**d**)) directions are overlaid.

Figure 5a shows the ARPES intensity map in the *k_x_*–*k_y_* plane, which was obtained integrating spectral intensity over −0.1 eV to 0 eV. One can see clearly a six-fold symmetric intensity distribution in Figure 5a, which is consistent with the symmetry of the MnTe(0001) surface. The surface/bulk Brillouin zone is shown in Figure 5b. According to previous *k_z_* dependent measurements [29], the $\bar{\Gamma }$ point of the surface Brillouin zone taken at *hν* = 117 eV is closer to the A point of the bulk Brillouin zone. Our ARPES results are consistent with previously reported ARPES results on bulk single crystals of cleaved surface [29].

Figure 5c,d exhibit ARPES image plots along the $\bar{\Gamma M}$ and $\bar{\Gamma K}$ directions, respectively. We have overlaid the band points given by the DFT + *U* along the AL (green circles in Figure 5c) and AH (blue circles in Figure 5d) directions of the bulk Brillouin zone. Since MnTe is typically hole-doped, the energy scale in the DFT + *U* calculations is referenced to the top of the valence band located at the A point. The shape of the convex band dispersion with its maximum at the A point in the DFT + *U* calculations is consistent with the ARPES image plots. The good agreement between theory and experiment confirms that a well-defined MnTe(0001) surface has been successfully prepared.

To examine the experimental band points in more detail, we fitted the momentum distribution curves (MDCs: ARPES intensity as a function of wavenumber at a given energy) using a single Lorentzian for *k*_//_ < 0 Å^−1^ and from −0.8 eV up to −0.1 eV. The extracted experimental band points are represented by open circles in Figure 5c,d. We limited the fitting region because the spectral intensity is stronger for *k*_//_ < 0 Å^−1^, and peak fitting was not stable above −0.1 eV and below −0.8 eV due to significant peak broadening. Subsequently, we fitted the experimentally obtained band points within the energy range from −0.6 eV up to −0.1 eV using a linear function, as indicated by the red lines in Figure 5c,d. Then, the Fermi wave number was estimated as *k*_F_ ≈ ±0.1 Å^−1^. To achieve agreement with this experimental *k*_F_ value, the theoretical band dispersion should be shifted toward higher energies (hole doping direction) by approximately 0.1 eV. As the intensity of the dispersive spectral features is significantly suppressed near the valence band maximum, one cannot see well-defined Fermi surfaces due to hole doping, as shown in Figure 5a.

Considering the value of the *c*/*a* ratio, the local MnTe_6_ octahedral structure is close to the ideal octahedral coordination. Consequently, Mn 3d orbitals directed toward the neighboring Te atoms are classified as e_g_ orbitals, while those oriented along directions toward next-nearest-neighbor Mn sites correspond to the t_2g_ orbitals. Significant p–d hybridization primarily occurs between the Mn 3d e_g_ orbitals and Te 5 p orbitals since Mn atoms have Te atoms as their nearest neighbors. In contrast, the Mn 3d t_2g_ orbitals, extending toward next-nearest-neighbor Mn atoms, form a narrow peak structure in the density of states, reflecting their localized character and thus contributing to the local magnetic moments. 

Recent ARPES measurements [26,27,28,29] have mainly focused on the band dispersions above −3 eV, specifically above the flat bands derived from Mn 3d t_2g_ states. Significant electron correlation effects are not visible because the dispersive bands near the valence band maximum predominantly originate from itinerant Te 5p states. Nevertheless, the existence of satellite structures due to electron correlation has been demonstrated in the wide energy range [33,34,35,39,41]. To further investigate electron correlation effects, we examine the occupied and unoccupied Mn 3d-derived states using resonant photoemission and inverse-photoemission spectroscopies.

**Figure 6:**
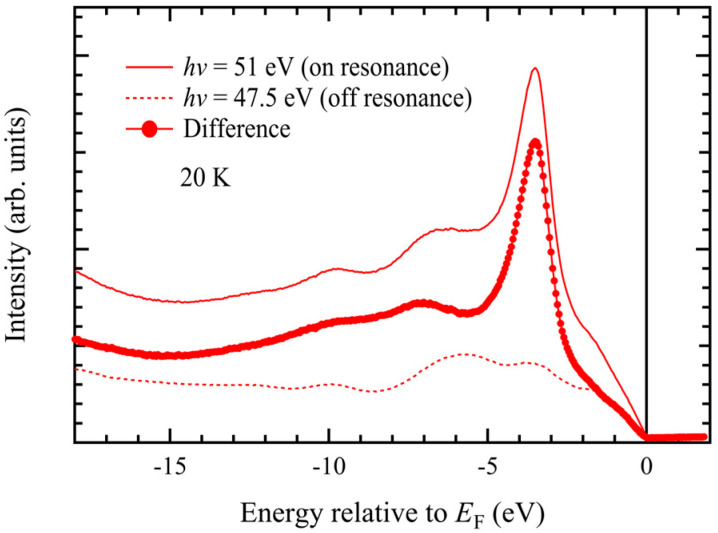
Resonant photoemission spectra taken at *hν* = 51 eV (on-resonance) and *hν* = 47.5 eV (off-resonance). The difference spectrum reveals the Mn 3d spectral intensity contribution.

Figure 6 shows angle-integrated photoemission spectra measured in the Mn 3p–3d resonance region under on-resonance (*hν* = 51 eV) and off-resonance (*hν* = 47.5 eV) photon-energy conditions. The main peak located around −3.5 eV is significantly enhanced in the on-resonance spectrum. The difference between the on- and off-resonance spectra in Figure 6 highlights the Mn 3d-derived spectral intensity. While the electron-correlation-derived satellite structure previously observed in polycrystalline MnTe appeared as a broad feature around −8 eV [33,34,35,39], we observed two distinct peaks at approximately −7 eV and −10 eV. Although the valence-band maximum is primarily derived from Te 5p states, we found a contribution from Mn 3d states from −2 eV up to the Fermi level.

**Figure 7:**
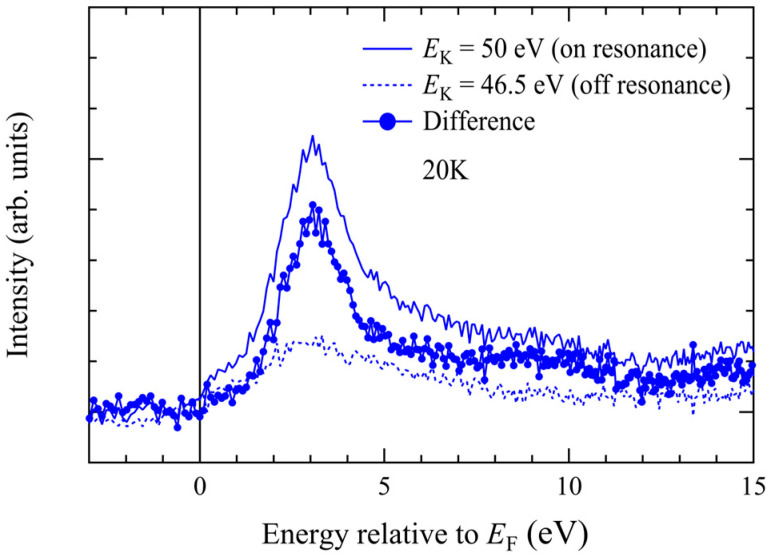
Resonant inverse-photoemission spectra measured at incident-electron-kinetic energies of *E*_K_ = 50 eV (on-resonance) and 46.5 eV (off-resonance). The difference spectrum reveals the Mn 3d spectral intensity contribution.

Figure 7 shows results of Mn 3p–3d resonant inverse-photoemission spectroscopy on MnTe(0001). At the on-resonance electron kinetic energy, the peak at +3.0 eV significantly enhanced, clearly indicating that it is derived from the Mn 3d↓ state. The spectral shape is similar to previous results on polycrystalline MnTe [36].

**Figure 8:**
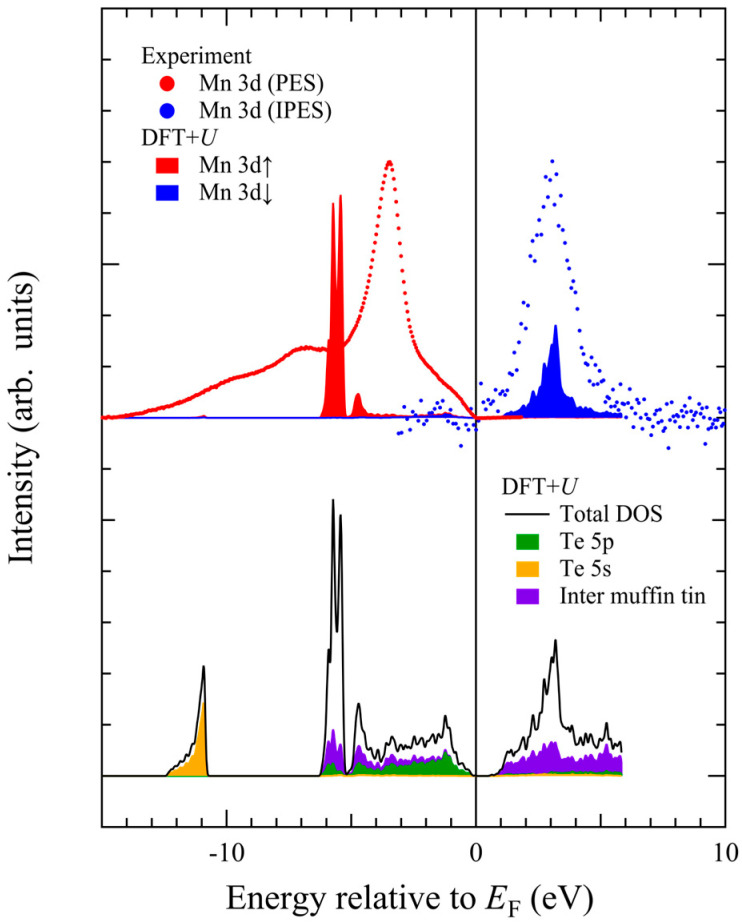
Theoretical partial density of states (DOS) inside the muffin-tin potential, as obtained from our DFT + *U* calculations: Mn 3d↑ partial DOS (red area), Mn 3d↓ partial DOS (blue area), total DOS (black line), Te 5p partial DOS (green area), Te 5s partial DOS (yellow area), and the DOS in the interstitial (inter muffin-tin) region (violet area). Experimentally determined occupied Mn 3d states (red circles) and unoccupied Mn 3d states (blue circles) are also shown.

In Figure 8, we show the theoretical partial density of states (DOS) inside the muffin-tin potential given by our DFT + *U* calculations: Mn 3d↑ partial DOS (red area), Mn 3d↓ partial DOS (blue area), total DOS (black line), Te 5p partial DOS (green area), and Te 5s partial DOS (yellow area). Note there exist electronic states in the inter muffin-tin region (violet area).

The Mn 3d-t_2g↑_-derived DOS in the occupied state exhibits a narrow peak at around −5.5 eV, and the Mn 3d-t_2g↓_-derived DOS in the unoccupied state has a peak at +3.5 eV. The Te 5p states extend from −6 eV up to the top of the valence band maximum. The contribution from Te 5p partial DOS is dominant from −4 eV up to the Fermi level. Note that there are broad states in the inter muffin-tin region forming almost flat DOS from −6 eV up to +6 eV. As the Te 5p states are primarily located in the occupied states, the unoccupied Mn 3d↓ states are energetically separated from the Te 5p states by the band gap. 

The upper panel of Figure 8 shows the Mn 3d-derived spectra obtained via resonant photoemission and inverse-photoemission spectroscopies after background subtraction using Shirley’s method [53]. The observed Mn 3d↑ is located at −3.5 eV in the photoemission spectra, and Mn 3d↓ is located at +3.0 eV in the inverse-photoemission spectra. The experimental *U*_eff_ value is 6.5 eV, which is consistent with previous results [33,34,35,36].

It is now clear that the experimentally observed Mn 3d↑ energy position significantly deviates from the DFT + *U* calculation, whereas the observed Mn 3d↓ energy position agrees well. The theoretical peak separation between Mn 3d↑ and Mn 3d↓ amounts up to 9 eV, which is about 2.5 eV larger than the experimental value. If we set *U* = 0 eV, the peak separation reduces to 4 eV [37,38], but the band gap disappears. Although theoretical Mn 3d peak positions could be adjusted by *U* values, the ground-state properties such as magnetic moment are well reproduced by *U*_eff_ ~ 4–5 eV [14,47]. In addition, it is impossible to reproduce the observed satellite structure in the gap region between −10 eV and −7 eV within the DFT + *U* scheme. 

Although the photoionization cross sections of the Te 5s and Te 5p orbitals are more than one order of magnitude smaller than that of Mn 3d around *hν* ~ 50 eV [54], the off-resonance spectrum in Figure 6 primarily reflects these states. The Te 5s states are located around −12 eV, and the Te 5p states extend from −7 eV up to the Fermi level. These states have been more clearly identified in the X-ray photoemission spectrum [41]. 

## 4. Discussion

**Figure 9:**
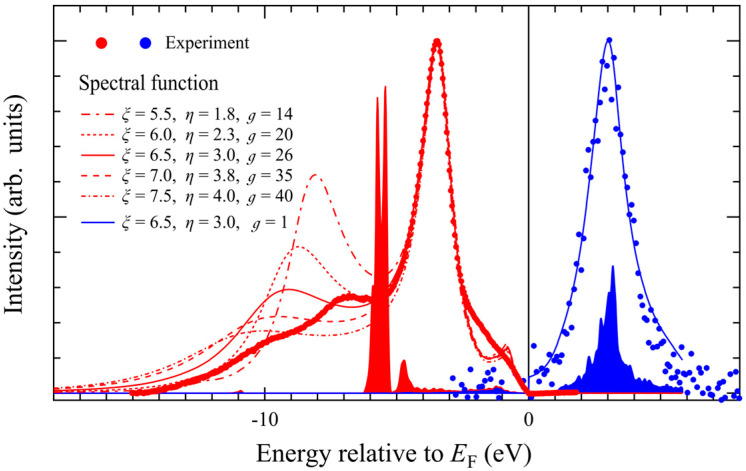
Spectral function calculated using a model self-energy. Parameter values are listed in the legend. Experimental data points (filled circles) and DFT + *U* results for Mn 3d↑ (red area) and Mn 3d↓ (blue area) are also shown. To account for impurity/defect scattering effects and instrumental energy resolution, energy-independent lifetime broadenings of 0.2 eV (full width at half maximum, FWHM) and 0.8 eV (FWHM) were applied to the occupied and unoccupied states, respectively.

While GW calculations generally provide more accurate descriptions of quasiparticle properties compared to DFT + *U*, a previous study reported that GW band dispersions for MnTe are qualitatively similar to those obtained from DFT + *U* calculations [55]. In this study, therefore, we have assumed that the DFT + *U* can provide reasonable ground state properties, and we introduce *k*-independent self-energy to simulate Mn 3d-derived spectral function. Figure 9 shows spectral functions with various *ξ*, *η*, and g values. While the simulated spectral intensity for the satellite structure is stronger, we can reproduce the peak shift and appearance of the satellite structure, which cannot be explained by DFT + *U* calculations. Based on the fitting, the excitation energy *ξ* was estimated to be in the range of 6–7 eV, which is comparable with the experimental *U*_eff_ value. It suggests that the spectral features in the occupied region reflect charge excitations of the order of 6–7 eV.

On the other hand, for the unoccupied region, the observed Mn 3d↓ peak coincide with the DFT + *U* results. In Figure 9, we used *ξ* = 6.5 eV with a much reduced g value (intensity of the self-energy) to reproduce experimental results. It indicates significant difference of the dynamical relaxation in the hole addition process and electron addition process.

We assume this difference originates from the difference in the relaxation processes. For the occupied states, the Te 5p states are located near the Mn 3d↑ states. Therefore, there exist more relaxation channels via the p–d hybridization in the hole-addition process. One can interpret the peak at −3.5 eV in the spectral function as the final state relaxed by the p−d hybridization, likely corresponding to the $d^{4}\underline{L}$ final states in the CI cluster model calculation. Since the Te 5p states in MnTe are almost completely filled, the Te 5p contribution is small in the unoccupied states. Furthermore, the unoccupied Mn 3d↓ states are isolated by the band gap. We assume that the relaxation in the electron-addition process is, therefore, suppressed compared to the hole-addition process.

If we calculate the coupling parameter of the many-body interactions in Mn 3d states, we obtain λ=0.4−0.5 , which is intermediate between λ≈0 (weakly correlated) and λ>1 (strongly correlated). The effective mass enhancement factor is calculated to be $m^{*}/m_{b}=1+\lambda =1.4-1.5$ , and the renormalization factor is z=1/(1+λ)=0.67−0.71 . Since the valence-band maximum of pristine MnTe is predominantly derived from Te 5p states, electron correlation effects are expected to be weak. However, the substitution of Mn and/or Te with elements that enhance the contribution of d-orbitals near the valence band maximum could lead to pronounced correlation effects.

## 5. Conclusions

We have investigated the occupied and unoccupied electronic states of an altermagnetic MnTe single crystal using photoemission and inverse-photoemission spectroscopies. We have established a reproducible procedure for obtaining a clean MnTe(0001) surface through repeated sputtering and annealing cycles. The angle-resolved photoemission spectroscopy taken at *hν* = 117 eV exhibited a hole-like band dispersion, which is consistent with our DFT + *U* calculations along the AL and AH directions. The observed Mn 3d↑-derived peak at −3.5 eV, however, significantly deviated from the DFT + *U* calculations, whereas the Mn 3d↓-derived peak observed by inverse-photoemission spectroscopy agreed well with the DFT + *U* results. Based on simulations of the spectral function employing an *ω*-dependent self-energy, we found significant relaxation effects in the electron-removal process, whereas such effects were suppressed in the electron-addition process. Our study provides a comprehensive picture of the electronic states, forming a solid foundation for understanding the magnetic and transport properties of MnTe. 

## Data Availability

The original contributions presented in this study are included in the article. Further inquiries can be directed to the corresponding authors.

## Abbreviations

The following abbreviations are used in this manuscript:

ARPES	Angle-resolved photoemission spectroscopy
CI	Configuration Interaction
DFT	Density Functional Theory
DOS	Density of States
EPMA	Electron Probe Microanalysis
GGA	Generalized Gradient Approximation
IPES	Inverse-photoemission spectroscopy
MDC	Momentum Distribution Curve
PES	Photoemission spectroscopy
SEM	Scanning Electron Microscope
SOC	Spin–Orbit Coupling
XRD	X-ray Diffraction
